# Endothelin‐1 rs9296344 associates with the susceptibility of childhood primary nephrotic syndrome

**DOI:** 10.1002/jcla.23134

**Published:** 2020-01-25

**Authors:** Ruifeng Zhang, Huandan Yang, Bingbing Zhu, Tingting Yuan, Qianqian Peng, Juan Lv, Shan Qiu, Suqin Zhou, Yan Li, Zhaowen Zhong

**Affiliations:** ^1^ Xuzhou Children's Hospital Xuzhou China

**Keywords:** childhood primary nephrotic syndrome, *EDN1*, SNP

## Abstract

**Background:**

Recently, the rs5370 single nucleotide polymorphisms (SNPs) of *Endothelin‐1* (*EDN1)* showed association with the susceptibility of childhood primary nephrotic syndrome (CPNS). This study aims to investigate potential relationships between other *EDN1* SNPs and CPNS.

**Methods:**

Seven SNPs (rs5370, rs10478723, rs1476046, rs1800541, rs2070698, rs2071942, and rs9296344) of the *EDN1* gene were genotyped in 579 CPNS patients and 586 age‐matched healthy children. Then, we analyzed potential associations of the six SNPs with susceptibility of CPNS by using rs5370 as a conditional variant in a logistic regression model. SNP‐SNP interaction analysis was performed to investigate the joint effects of the seven SNPs in the pathogenesis of CPNS.

**Results:**

Independent with rs5370, only rs9296344 significantly associated (T vs C, odds ratio [OR] = 0.71, 95% confidence interval [CI] = 0.57‐0.88, *P* = .001) with the susceptibility of CPNS. Meanwhile, no joint effect among the analyzed seven SNPs was discovered in this study.

**Conclusions:**

This study discovered that C allele of rs9296344 on *EDN1* is a novel independent risk factor for CPNS.

## 1 INTRODUCTION

Childhood nephrotic syndrome is a group of symptoms that indicate kidney damage, particularly damage to the glomeruli, the tiny units within the kidney where blood is filtered, and results in the release of too much protein from the body into the urine.^1^, ^2^ Childhood primary nephrotic syndrome (CPNS) is the most common type of childhood nephrotic syndrome.^1^, ^3^ There are around 16 ~ 18 CPNS patients in every 100 000 children.^4^ The cause of CPNS is not known in most cases. However, recent studies suggested that genetic factors, such as single nucleotide polymorphisms (SNPs), might contribute to the susceptibility of CPNS.^5^, ^6^, ^7^, ^8^

*Endothelin‐1 (EDN1)* encodes a preproprotein that is proteolytically processed to generate a secreted peptide that belongs to the endothelin/sarafotoxin family. This peptide is a potent vasoconstrictor, and its cognate receptors are therapeutic targets in the treatment of pulmonary arterial hypertension. The overexpression of *EDN1* associated with pathological kidney phenotypes, such as age‐dependent development of renal cysts, interstitial fibrosis of the kidneys, and glomerulosclerosis, and leads to a progressive decrease in glomerular filtration rate.^9^ Fang Yang et al^5^ genotyped three SNPs in *EDN1* and discovered the association of rs5370 with the clinical phenotype in CPNS. Mohammad Hashemi et al^10^ further confirmed the association of *EDN1* rs5370 G > T gene polymorphism with the susceptibility of CPNS in Iranian population. However, there are more than three SNPs with a minor allele frequency (MAF) over 0.1 in *EDN1*; it is still unknown whether other SNPs in *EDN1* are associated with the susceptibility of CPNS independent of rs5370*.*

To study the potential rs5370‐independent relationships between other SNPs of *EDN1* and susceptibility of CPNS, we genotyped seven SNPs (rs5370, rs10478723, rs1476046, rs1800541, rs2070698, rs2071942, and rs9296344) of *EDN1* and used rs5370 as conditional variant in the regression model to analyze the potential association independent of rs5370.

## 2 MATERIALS AND METHODS

### 2.1 Participants

Childhood primary nephrotic syndrome patients and age‐matched healthy child participants were enrolled in between July 1, 2014, and October 31, 2018, in Xuzhou Children's Hospital. All participants are in Chinese Han population. A full rationalization of the procedure was conducted to legal guardians of all participants to obtain written consent. The performance of this study was approved by the ethics committee of Xuzhou Children's Hospital following the principle of the Helsinki Declaration.

### 2.2 Polymerase chain reaction (PCR) and DNA sequencing

Blood samples were drawn from CPNS patients and age‐matched healthy child participants to extract DNA with the QIAamp DNA Blood Mini Kit (Qiagen). PCR was used to capture selected SNPs from DNA. PCR system for 50 μL was as follows: The reaction mixture contained 2 μL DNA, 2 μL primers, 4 μL dNTP, 5 μL PCR buffer, and 0.25 μL Taq enzyme (Takara), and then, the water was added to make up a volume of 50 μL. The reaction conditions were set as follows: 94°C for 5 minutes; 39 amplification cycles, 30 seconds at 94°C, 45 seconds at 53.7°C, and 50 seconds at 72°C; and ultimate extension lasted for 5 minutes at 72°C. All the six SNPs (rs10478723, rs1476046, rs1800541, rs2070698, rs2071942, and rs9296344) in *EDN1* were selected with minor allele frequencies (MAF) >0.1 in Chinese population according to SNP data in dbSNP database (http://www.ncbi.nlm.nih.gov/SNP). Among the six SNPs, rs9296344 locates on the 3'UTR region, while the others locate on intron region. The sequences of PCR products were obtained by using an ABI PRISM 3730 genetic analyzer (Applied Biosystems).

### 2.3 Statistical analysis

The PLINK software was used to determine the association of single SNP loci in genotype and allele frequencies with CPNS, as well as the *P‐value* of the Hardy‐Weinberg equilibrium (HWE). The rs5370 was used as conditional variant in the regression model to analyze the potential associations independent of rs5370. The risk of alleles was estimated by the odds ratio (OR) with 95% confidence intervals (CIs). Linkage disequilibrium (LD) between SNPs was measured by D' and *R* ^2^ using Haploview software. *P*‐values are two‐tailed with the threshold of .05 for statistical significance. All statistical analyses were conducted by using R (version 3.5.2). The SNP‐SNP interaction was performed by using GMDR software,^11^ which is the software for detecting gene‐gene and gene‐environment interactions underlying complex traits. *P‐value *<.05 was selected as significant results.

## 3 RESULTS

### 3.1 C allele of rs9296344 associates with the susceptibility of CPNS

There were 579 CPNS patients (272 boys and 307 girls with a mean age ± SD of 8.56 ± 4.01 years) and 586 age‐matched healthy children (277 boys and 309 girls with a mean age ± SD of 8.80 ± 4.10 years) involved in this study (Table 1). The SNP rs5370 and other six SNPs (rs10478723, rs1476046, rs1800541, rs2070698, rs2071942, and rs9296344) with a MAF over 0.1 in Chinese population in dbSNP database were genotyped in blood samples of both CPNS and healthy participants. The rs5370 was used as the conditional variant in the regression model to analyze the potential SNP‐CPNS associations independent of rs5370. The distribution of the genotypes of all the six SNPs was in the Hardy‐Weinberg equilibrium (HWE) (HWE *P‐value *>.001) in all the healthy children. In CPNS patients, rs9296344 is the only one that did not in the HWE (HWE *P‐value *<.001). Moreover, the allele frequencies and genotype frequencies of rs9296344 were significantly (*P‐value *<.05) associated with CPNS after removing the effect of rs5370 using conditional analysis (Table 2). The C allele frequency of rs9296344 was higher in CPNS patients than in healthy children (T vs C, odds ratio [OR] = 0.71, 95% confidence interval [CI] = 0.57‐0.88, *P* = .001). It revealed that C allele of rs9296344 associates with the susceptibility of CPNS. Meanwhile, we did not find significant associations in allele frequencies or genotype frequencies of the other five SNPs with the risk of CPNS.

**Table 1:** Clinical features of participants

	Patients	Control
Population size	579	586
Age (years)	8.56 ± 4.01	8.80 ± 4.10
Male	272	277
Female	307	309

**Table 2:** Alleles and genotypes of analyzed SNPs in studied populations

Group	Allele frequency		*P*‐value (rs5370 as conditional variant)	OR	95% CI	Genotype frequency			*P*‐value	HWE
rs10478723	G	A				GG	GA	AA		
Patient	0.85579	0.14421	.540995	0.929583	0.736‐1.175	0.72949	0.27051	0.01047	.437172	0.0419
Control	0.86457	0.13543				0.75606	0.24394	0.01557		0.533368
rs1476046	G	A				GG	GA	AA		
Patient	0.78152	0.21848	.634187	0.953115	0.782‐1.162	0.6277	0.3723	0.04137	.36359	0.258985
Control	0.78961	0.21039				0.66129	0.33871	0.05197		0.453568
rs1800541	T	G				TT	TG	GG		
Patient	0.74352	0.25648	.178641	0.878384	0.727‐1.061	0.57117	0.42883	0.05657	.320275	0.303423
Control	0.76746	0.23254				0.6147	0.3853	0.05197		0.526056
rs2070698	T	C				TT	TC	CC		
Patient	0.50604	0.49396	.739102	1.027972	0.874‐1.209	0.29933	0.70067	0.28381	.661588	0.027353
Control	0.49915	0.50085				0.3139	0.6861	0.31614		0.302103
rs2071942	G	A				GG	GA	AA		
Patient	0.78497	0.21503	.781627	1.028226	0.845‐1.252	0.66484	0.33516	0.06044	.955317	0.125093
Control	0.78024	0.21976				0.65642	0.34358	0.06148		0.17376
rs9296344	T	C				TT	TC	CC		
Patient	0.79706	0.20294	.001429	0.706584	0.57‐0.875	0.71881	0.28119	0.07821	3.82E‐05	3.09E‐06
Control	0.84753	0.15247				0.73647	0.26353	0.02443		0.909996

Abbreviation: SNP, single nucleotide polymorphism.

### 3.2 Linkage disequilibrium (LD)

**Figure 1:**
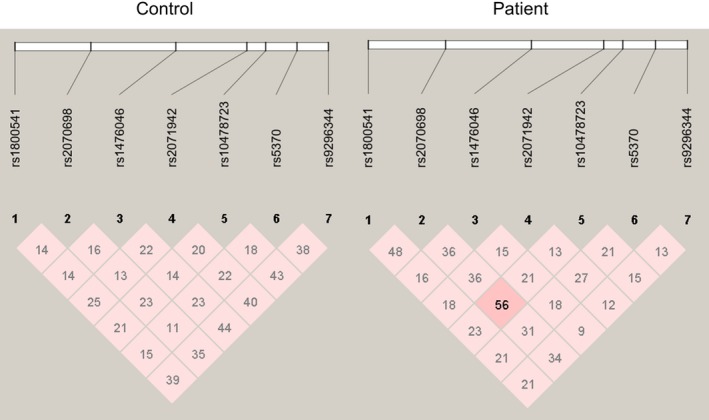
Linkage disequilibrium analysis of analyzed SNPs in patients and healthy controls

Linkage disequilibrium analysis of the seven SNPs in CPNS patients and age‐matched healthy children revealed that only weak (D' <0.4) or median LD (0.4 < D' <0.6) interactions existed in these analyzed SNPs (Table 3, Figure 1). No strong LD (D' >0.6) interactions were discovered among these SNPs, which means that these SNPs act independently in both CPNS patients and age‐matched healthy child population. Therefore, we did not perform further haplotype analysis in this study.

**Table 3:** The linkage disequilibrium analysis of analyzed SNP pairs in studied populations

SNP1	SNP2	Patients			Controls		
		D'	LOD	R2	D'	LOD	R2
rs1800541	rs2070698	0.488	21.8	0.084	0.143	4.62	0.019
rs1800541	rs1476046	0.16	4.93	0.021	0.147	2.97	0.013
rs1800541	rs2071942	0.184	6.37	0.027	0.256	14.09	0.061
rs1800541	rs10478723	0.235	6.2	0.027	0.21	6.7	0.03
rs1800541	rs5370	0.206	7.53	0.031	0.151	4.81	0.02
rs1800541	rs9296344	0.21	7.63	0.033	0.399	10.98	0.042
rs2070698	rs1476046	0.367	9.88	0.039	0.161	3.36	0.014
rs2070698	rs2071942	0.365	9.57	0.037	0.133	4.2	0.018
rs2070698	rs10478723	0.562	14.39	0.055	0.236	8.03	0.035
rs2070698	rs5370	0.32	12.64	0.05	0.117	3.07	0.013
rs2070698	rs9296344	0.344	7.88	0.031	0.354	9.12	0.035
rs1476046	rs2071942	0.157	5.65	0.024	0.224	6.28	0.028
rs1476046	rs10478723	0.218	6.46	0.029	0.14	3.8	0.017
rs1476046	rs5370	0.173	4.3	0.018	0.23	6.28	0.028
rs1476046	rs9296344	0.096	2.01	0.008	0.445	8.09	0.031
rs2071942	rs10478723	0.13	2.43	0.01	0.206	6.1	0.027
rs2071942	rs5370	0.266	9.84	0.041	0.229	11.52	0.049
rs2071942	rs9296344	0.127	3.54	0.015	0.406	12.14	0.047
rs10478723	rs5370	0.197	3.32	0.014	0.181	4.51	0.019
rs10478723	rs9296344	0.151	3.45	0.015	0.436	8.87	0.034
rs5370	rs9296344	0.116	1.77	0.007	0.38	11.27	0.043

Abbreviation: SNP, single nucleotide polymorphism.

### 3.3 SNP‐SNP interaction analysis

single nucleotide polymorphism‐SNP interaction analysis was performed to investigate the joint effects of the seven SNPs (including rs5370) in the pathogenesis of CPNS. Multiple model types ranging from 1 SNP locus to 7 SNP loci were investigated in this study. Models with best testing balanced accuracy in every loci size all had rs9296344. But no significant joint effects were discovered in this study (Table 4). This revealed that rs9296344 may play its role in CPNS independently.

**Table 4:** Comparison of the best models in GMDR analysis

Best model	Training balanced accuracy	Testing balanced accuracy	Sign test (*P‐value*)	Cross‐validation consistency
rs5370	0.6381	0.5853	4 (*P* = .3281)	8/10
rs5370 rs9296344	0.7352	0.6965	5 (*P* = .2230)	8/10
rs1476046 rs5370 rs9296344	0.6279	0.5853	4 (*P* = .5232)	6/10
rs2070698 rs1476046 rs5370 rs9296344	0.6153	0.5963	5 (*P* = .3231)	9/10
rs10478723 rs1476046 rs2070698 rs5370 rs9296344	0.5898	0.4863	2 (*P* = .7895)	8/10
rs10478723 rs1476046 rs1800541 rs2070698 rs5370 rs9296344	0.6457	0.5397	4 (*P* = .7282)	9/10
rs10478723 rs1476046 rs1800541 rs2070698 rs2071942 rs5370 rs9296344	0.6875	0.5972	4 (*P* = .8185)	10/10

## 4 DISCUSSION

Childhood primary nephrotic syndrome (CPNS) is the most common type of childhood nephrotic syndrome. It is a collection of symptoms due to kidney damage, including protein in the urine, low blood albumin levels, high blood lipids, and significant swelling.^1^, ^4^ Single nucleotide polymorphisms (SNPs) of genes have been found to contribute to the pathogenesis of many diseases,^12^, ^13^, ^14^, ^15^, ^16^, ^17^, ^18^ as well as CPNS 5, 10. Fang Yang et al^5^ reported the association between rs5370 and CPNS. Mohammad Hashemi et al further confirmed that rs5370 G > T gene polymorphism increases the risk of CPNS in Iranian population.^10^ This time, by removing the effect of rs5370 using conditional analysis, we discovered C allele of SNP rs9296344 (T vs C, odds ratio [OR] = 0.71, 95% confidence interval (CI] = 0.57‐0.88, *P* = .001) of the *EDN1* gene significantly associated with the susceptibility of CPNS.

*Endothelin‐1* encodes a preproprotein, which associated with pathological kidney phenotypes, such as age‐dependent development of renal cysts, interstitial fibrosis of the kidneys, and glomerulosclerosis, and leads to a progressive decrease in glomerular filtration rate.^9^ The association of *EDN1* rs5370 G > T gene polymorphism revealed the potential relationship between *EDN1* gene polymorphism and the pathogenesis of CPNS. In this study, we discover a novel susceptibility SNP rs9296344 in 579 CPNS patients and 586 healthy controls of Chinese Han population, which is a larger population than previous studies.^5^, ^10^ The discovery of new susceptibility SNP revealed that *EDN1* polymorphisms truly associated with the susceptibility of CPNS and that vasoconstrictor may act roles in the pathogenesis of CPNS.

Unlike the missense variant rs5370, which will change the sequence of EDN1 protein, the SNP rs9296344 locates on the 3'UTR region of *EDN1* gene, which would not change the sequence of EDN1 protein. But 3'UTR plays important roles in the regulation of gene expression.^19^, ^20^ The association of rs9296344 C allele implied that it may affect the expression of *EDN1* gene, since overexpression of *EDN1* is associated with pathological kidney phenotypes in CPNS.^9^ A potential mechanism is that the rs9296344 C allele may increase the susceptibility of CPNS by increasing the expression of *EDN1.*

In general, this study reported that rs9296344 C allele on *EDN1* associates with the susceptibility of CPNS. The discovery of novel susceptibility SNP confirmed the role of *EDN1* in the pathogenesis of CPNS and promoted novel diagnostic and therapeutic technologies for CPNS.
